# Medical and Physical Intelligence

**Published:** 1803-06-01

**Authors:** 


### MEDICAL AND PHYSICAL INTELLIGENCE.

### [ FOREIGN AND DOMESTIC. ]

---

*A successful Method of treating the Yellow Fever at its Commencement; communicated, May* 25, 1803, *by* Dr. HARNESS, *Commissioner for Sick and Wounded Seamen.*

LIEUT. DOUGLAS, of the 85th regiment, relates, that he embarked on board the Chichester store-ship at Jamaica for England with one hundred and eighty men, seventy-four of whom died on the passage previous to reaching Hallifax in North America, exclusive of the Captain, two Lieutenants, Surgeon, and Surgeon's Mate of the Ship. In consequence of the two latter having fallen victims to the disease, Lieut. Douglas felt himself driven to the necessity of undertaking the treatment of the sick; and from the great fatality attendant on the calomel and purgative plan, pursued by the late Surgeon and his Mate, he (Lieut. D.) was induced to adopt Bleeding, (as recommended by Dr. Jackson, and as had been suggested in Lieut. D's presence by the Surgeon's Mate of the 60th regiment, a short time previous to Lieutenant D. leaving Jamaica) which proved to have been productive of the happiest effects, as will evidently appear by the following statement.

Lieut. D. relates, that after the care of the sick had devolved upon him, sixty-two men (37 of whom were seamen) were attacked with symptoms of yellow fever, the whole of whom recovered by bleeding; three others were likewise bled, but he observes, so late in the disease, or not until the symptoms of fever were so fully established, as not to be within reach of the remedy.

Lieut. D. remarks, the success in treating the disease was so evident to the troops and ship's company, that after a short time they would, on being taken ill, apply to be bled; and Lieut. D. became so confident of its good effects, if had recourse to at the onset of the disease, as to induce him to give particular directions to be called any hour of the night to perform the operation, should any one be seized with the leading symptoms of the disease; and in every every case (the three alluded to excepted) he had the happiness to see every symptom give way or diminished; and all unfavourable appearances removed by one, two, or three repeated bleedings performed at intervals of a few hours, as the necessity of the remaining symptoms indicated.

Lieut. D. not being educated to the profession, and consequently ignorant of the doses of medicines, was induced to have recourse to clysters, when the procuring of evacuations appeared necessary; on which, with bleedings as before mentioned, he rested the whole means of cure.*

---

Among the new Memoirs of the Academy of Sciences of Stockholm, (1801) the Essay of M. Acharius, *on the Use and Advantages of Tar-Water in Venereal Complaints*, is chiefly remarkable. The author insists that this water is often sufficient to operate a cure, and that there is no necessity to strengthen its operation by mercury, the fatal consequences of which are so well known; and that even if its efficacy were not sufficient, it is, at least, always an excellent preparative, calculated to facilitate wonderfully the effects of the mercury, and to diminish the doses of it. M. Acharius prepares his liquor cold; by this means the tar loses less of its acid; and as it is to serve for drink, he mixes it with common water, in the proportion of forty-five parts of water to two of tar. In the compressions or bandages applied to ulcers, &c. one part of tar should be dissolved in fifteen parts of water; the *balnea* or lotions administered two or three times a week have greatly contributed to accelerate the cure. Many patients have been compleatly cured by the external and internal use of this water alone, while others have found its salutary effects when combined with mercury or other remedies. Out of seventy patients, only eighteen were obliged to have recourse to the use of mercury with tar-water. The above-cited Essay enumerates the cases of a number of patients cured at the hospital of Stockholm, by the use of this specific only.

---

A letter from Dr. C. Caldwell of Philadelphia, to Dr. Patterson, in Londonderry, contains the following observations:

"On the score of science I have nothing particularly interesting to communicate to you. I think, however, our Philosophical Society

---

* Our admiration of the uncommon merit of this officer, naturally led us to enquire whether his conduct had been properly represented to those who have the power to reward it, and we were assured that it had. We also learned, that apprehending the duties of captain and master of the ship might possibly devolve on him, as well as those of the surgeon and mate, he devoted himself to the study of navigation, and in a short time made such a progress in the practical part of it, as would have enabled him to conduct the ship home with safety. We hope this note will induce his friends to gratify us with more particular information respecting him, and his future promotion. Ed.

ciety is becoming more than usually ardent and active in pursuit of the objects and ends of its institution. Dr. Woodhouse is going on with a series of experiments on the oxyd, and other preparations of carbon, which promise to throw light on Chemistry, and perhaps to give to the Lavoisierian doctrine a more stunning, not to say fatal blow, than it has ever before received. His Memoir, when compleated, will probably be sent for publication to some of the European periodical works. Dr. Barton has in the press two works of some magnitude, viz. "The Elements of Botany," and "Fragments of the Natural History of Pennsylvania," Part, or rather No. II. I am myself, (Dr. C. Caldwell) engaged in several minor points of inquiry. One of the most interesting of them is the vitality of the blood. From my experiments, hitherto made, on this subject, I am disposed to consider, with the learned leader of Israel, and say, that "the life is in the blood." But I have not yet conducted my experiments to a close, and must not therefore pronounce decisively."

---

Cit. Thenard proposes the following method of purifying rape-seed oil: Mix to one hundred parts of oil, two parts of concentrated sulphuric acid, and shake them well; after which the oil will immediately change its colour, and become dark green; and when it has been suffered to stand for about one hour, flakes begin to be deposited. Add now, by degrees, double its weight of water, for the purpose of properly diluting the sulphuric acid. The mixture having been stirred for half an hour is suffered to stand quietly during eight days, after which the oil will be found to swim on the water, which stands over a blackish matter, precipitated from the oil by means of the sulphuric acid, and it is this matter which prevents its burning properly, and to which the general colour of that oil is owing. Three strata therefore may be distinguished in this mixture; the first, that of oil; the second, of water mixed with a little sulphuric acid; and the third, of coal. If the oil is intended to be quite transparent, it ought to stand for a longer time than eight days; but this may be shortened by letting it pass through a filtrum of woollen cloth, by which means it is rendered perfectly clear. By this proceeding, when properly and carefully employed, an oil is obtained, which is very little tinged, without any smell, and quite tasteless, and which burns extremely well. The loss during the whole process is inconsiderable. This process may be pushed still farther, by mixing one hundred parts of the above purified oil with one part of concentrated sulphuric acid, which occasions a grey precipitate that is with difficulty separated from the oil; but on its being digested with 0,02 of sulphuric acid and with one-fourth of its weight of lime, or argilla, the oil is obtained as white as clear water. The loss of oil, however, is very considerable; the argilla is more advantageous than the lime, as it may be afterwards expressed.

---

A Cor-

A Correspondent in Paris has lately informed us, that Mr. Vauquelin has discovered phosphat of lime in several kinds of corn, which proves the analogy of the chemical processes in the animal body with those in the vegetable organization, as such a combination had been as yet only found in the bones of animals.

---

Another new metal has been discovered, which is called *palladium*, or new silver. It possesses the following properties. 1. It dissolves in pure spirit of nitre, and makes a dark red solution. 2. Green vitriol throws it down in a state of regulus from this solution, as it always does gold from *aqua-regia*. 3. If the solution be evaporated, a red calx is obtained, that dissolves in spirit of salt or other acids. 4. It is thrown down by quick-silver, and by all the metals, except gold, platina, and silver. 5. Its specific gravity, by hammering, was only 11.3; but by flatting, it is as much as 11.8. 6. In a common fire it tarnishes a little and turns blue, but comes bright again, like the other noble metals, when strongly heated. 7. The greatest heat of a blacksmith's fire would hardly melt it. 8. But if it be touched while hot with a small bit of sulphur, it runs as easily as zinc.

---

Dr. Bouttatz, Russian physician, lately in London, has been commissioned by the Emperor of Russia to travel through that empire for the purpose of extending the Vaccine Inoculation.

---

Dr. BATTY will commence his Summer Course of Lectures on the Theory and Practice of Midwifery, and the Diseases of Women and Children, on June the 20th, at nine o'clock in the morning, at his house in Great Marlborough Street.

